# Innate Immune Cells in Immune Tolerance After Liver Transplantation

**DOI:** 10.3389/fimmu.2018.02401

**Published:** 2018-11-09

**Authors:** Hongting Huang, Yefeng Lu, Tao Zhou, Guangxiang Gu, Qiang Xia

**Affiliations:** Department of Hepatic Surgery and Liver Transplantation Center, School of Medicine, Renji Hospital, Shanghai Jiaotong University, Shanghai, China

**Keywords:** liver transplantation, innate immune cells, immune tolerance, dendritic cells, Kupffer cells, NK cells, LSECs

## Abstract

Currently, liver transplantation is the most effective treatment for end-stage liver disease. Immunosuppressive agents are required to be taken after the operations, which have significantly reduced rejection rates and improved the short-term (<1 year) survival rates. However, post-transplant complications related to the immunosuppressive therapy have led to the development of new protocols aimed at protecting renal function and preventing *de novo* cancer and dysmetabolic syndrome. Donor specific immune tolerance, which means the mature immune systems of recipients will not attack the grafts under the conditions without any immunosuppression therapies, is considered the optimal state after liver transplantation. There have been studies that have shown that some patients can reach this immune tolerance state after liver transplantation. The intrahepatic immune system is quite different from that in other solid organs, especially the innate immune system. It contains a variety of liver specific cells, such as liver-derived dendritic cells, Kupffer cells, liver sinusoidal endothelial cells, liver-derived natural killer (NK) cells, natural killer T (NKT) cells, and so on. Depending on their specific structures and functions, these intrahepatic innate immune cells play important roles in the development of intrahepatic immune tolerance. In this article, in order to have a deeper understanding of the tolerogenic functions of liver, we summarized the molecular mechanisms of immune tolerance induced by intrahepatic innate immune cells after liver transplantation.

## Introduction

The past three decades have seen liver transplantation becoming a major treatment approach for end-stage liver disease. This is because of the dramatic improvement in survival after liver transplantation. Actually, improved use of post-transplant immunosuppression is a major factor. In order to prevent acute and chronic rejection, long-term immunosuppression is required to avoid severe acute and chronic rejection and graft loss. Up to now, the backbone of immunosuppression therapy remains calcineurin inhibitors (CNIs) mostly associated with steroids in the short-term and mycophenolate mofetil or mammalian target of rapamycin (mTOR) inhibitors. However, there are some post-transplant complications related to immunosuppressive therapy, such as renal injury, *de novo* cancer, dysmetabolic syndrome, and so on. In the future, immunosuppression will be more oriented, aiming to protect the graft from rejection and at reducing the risk of disease recurrence and complications related to immunosuppressive therapy, including promoting stable long-term immunological tolerance of the liver graft. The liver has been shown to be more tolerogenic than other solid organs, and most hepatic allografts can be accepted with low-dose immunosuppressive therapy. It can be exemplified by oral tolerance (which means the mucosal immune system maintains unresponsiveness to antigens that might induce unexpected immune responses) and portal venous tolerance (which means the induction of peripheral tolerance following portal venous delivery of most alloantigens). Other phenomena attributed to hepatic immune tolerance consist of persistent microbial infections and gastrointestinal tumor metastases in the liver (1). Besides this, the liver can induce tolerance for other transplanted organs and decrease the risk of rejection of associated organs, such as heart, kidney, skin, pancreas, and so on (2). There were plenty of signs that showed that the internal microenvironment of the liver could play a role in the development of immune tolerance after transplantation.

Liver is an organ with double blood supply, from the portal vein and the hepatic artery. Arterial and venous blood mix in the liver, resulting in low oxygen tension, low perfusion pressure, and slow and irregular blood flow within the hepatic sinusoids, which assist the intrahepatic cells and molecules to fully contact each other. Generally, the adaptive system in the liver includes humoral immunity and cell-mediated immunity. They are carried by two different lymphocytes (B cells and T cells), which recognize and respond to pathogens in antigen-specific ways. By contrast, the innate immune cells contained in the liver are quite different from those in the peripheral blood, including liver-derived dendritic cells (DCs), Kupffer cells, liver sinusoidal endothelial cells (LSECs), liver-derived natural killer (NK) cells, natural killer T (NKT) cells, and so on. These innate immune cells participate in constituting the immune microenvironment in the liver. Their tolerogenic functions are met by two mechanisms. First, intrahepatic innate immune cells express low or undetectable levels of major histocompatibility complex (MHC) antigens, costimulatory molecules, and other effector molecules, which means it will be difficult for these intrahepatic cells to induce innate or adaptive immune response. Second, innate immune cells in the liver can also exert their immunosuppressive effects by interfering with the functions of other intrahepatic cells, by secreting immunosuppressive cytokines [such as interleukin-10 (IL-10), transforming growth factor- β (TGF-β), indolamine 2,3-dioxygenase (IDO), and so on] or by directly contacting them. In this review, we clarified some potential mechanisms to illustrate human liver allograft tolerance induced by intrahepatic innate immune cells.

## Dendritic cells (DCs) in tolerance

Dendritic cells are derived primarily from the bone marrow, as well as from the liver and the spleen. As a heterogeneous population of antigen presenting cells (APCs), DCs play pivotal roles in the initiation of immunity and the induction of immunological tolerance depending on their maturation state and subsets. Recently, regulatory DCs, which mean DCs with immune regulatory functions, have attracted much attention. Up to now, several kinds of regulatory DC had been reported, such as CD11c^low^I-A^low^CD11b^hi^ splenic regulatory DCs (3) and CD11c^low^CD45RB^+^DCs (4, 5). They have common characteristics, including immature DC phenotype, high IL-10 and low IL-12p70 secretion, and inhibition of T-cell proliferation. It has been proved that the liver microenvironment could program differentiation of bone marrow derived progenitors into regulatory DCs. In a heterotopic liver transplantation model in rats, He et al. (6) have found that the graft survival rate can be greatly improved by infusing immature DCs (imDCs) into the recipient rats. It has been suggested that the overexpression of the zinc finger protein A20 could effectively inhibit the maturation of DCs that are resident in the liver allograft and consequently suppress acute liver allograft rejection. Dai et al. (7) have demonstrated that A20 treatment could significantly inhibit transplantation induced nuclear factor-κB (NF-κB) mediated activation of DCs resident in the liver allograft, which was consistent with the changes of costimulatory molecule and IL-12 mRNA expression of DCs. Since NF-κB has been shown as a pivotal nuclear transcriptional regulator of DC maturation and immunostimulatory ability (8), it presumably plays an essential role in this process.

In normal conditions, DCs derived from human or mouse livers can secrete certain amounts of IL-10, inducing lower T cell response and promoting regulator T cell (Treg) generation, with a definite effect on immune tolerance (9–11). Besides this, Kushwah et al. (12) have found that viable immature DCs have the ability to uptake apoptotic DC, which suppresses the subsequent maturation of viable DC and mediates the differentiation of naïve T cells into Foxp3^+^ Tregs (12). However, under inflammatory conditions, quiescent DCs transform from a tolerant state into a responsive state. Dendritic cells located in sinusoids and portal area can fully contact T cells and induce T cell-mediated immune responses (13).

## Myeloid dendritic cells (mDCs)

In mice, liver myeloid dendritic cells (mDCs) have lower maturity than those in secondary lymphoid tissues, both in terms of phenotype and function (14–16). It is mainly reflected by the lower expression level of MHC-II and costimulatory molecules on the cell surface (16) and lower secretion of IL-12 after toll-like receptor (TLR) activation while there is a higher production of IL-10 and IL-27, which have been found to be closely related to the low response of T cells induced by liver DCs (17). Overall, in *in vivo* experiments on mice, mDCs derived from the liver show reduced ability to activate allogeneic naïve T cells (14–16).

In addition to the lower maturation state, the liver-derived mDCs can also induce immune tolerance by affecting T cell function. In mice, Khanna et al. (18) injected liver-derived mDCs into allogeneic recipients and found that they can induce T cells to secrete IL-10 (18). Besides this, *in vitro* experiments have shown that the interactions between NK cells (through their inhibitory receptor NKG2A) and hepatocytes can change the concentrations of some important cytokines in the local microenvironment [for example, an increase in TGF-β and a decrease in tumor necrosise factor- α (TNF-α)], thus, inducing the differentiation of a group of special DCs that have an immune tolerance function. This kind of DCs can induce the differentiation of a special type of Treg, which can inhibit T cell response via the programmed death-1 (PD-1) pathway (19). Based on these results, Liu et al. (20) found that *in vitro* liver–derived DCs are more likely to induce Treg differentiation than spleen-derived DCs, and this process depended on the high programmed cell death-ligand 1 (PD-L1) expression level of liver-derived DCs (20).

Besides this, the interactions between mDCs and hepatic stellate cells (HSCs) can also induce immune tolerance effects. By secreting all-trans retinoic acid (ATRA), HSCs can induce the syntheses of arginase1 (ARG1) and inducible nitric oxide synthase (iNOS) in mDCs. Among them, ARG1 can degrade the arginine in the local microenvironment, while iNOS can induce the synthesis of NO, thus, inhibiting the function of effector T cells (21). At the same time, HSCs can secrete a variety of cytokines [for example IL-1, chemokine (C-C motif) ligand 2 (CCL2), and CCL3], which can activate the downstream signal transducer and activator of transcription 3 (STAT3) signaling pathway in mDCs and finally prompt mDCs to express IDO and PD-L1. The former (IDO) can catabolize the tryptophan in local microenvironment while the latter (PD-L1) can bind to the PD-1 on the effector T cells (22–24).

## Plasmacytoid dendritic cells (pDCs)

In general conditions, plasmacytoid dendritic cells (pDCs) can present a small fraction of antigens and then activate T cells, although its function is much weaker than mDCs. It has been found that when compared with spleen-derived pDCs, liver-derived pDCs in mice showed immature phenotype and low secretion level of IL-12p70; thus, they showed a reduced ability to present antigens or activate T cells (25, 26). Besides this, a new subpopulation of CCR9^+^ pDCs was identified as tolerogenic pDCs in an acute graft-vs.-host disease model (27). It has been demonstrated that this subset of pDCs exist abundantly in the murine liver, produce IL-10 and TGF-β, and induce the differentiation of naïve T cells into a regulatory phenotype through the TLR7/9 pathway (28).

In terms of the interactions with other cells, the liver-derived pDCs have a lower Delta4/Jagged1 ratio of Notch ligands on the cell surface, which means that they can predominantly induce T cells to differentiate into T helper 2 (Th2) cells. As with mDC, pDC can upregulate the expression of PD-L1 through the STAT3 pathway under the impact of IL-27, thereby, inhibiting the function of the effector T cell (29, 30).

## Kupffer cells in tolerance

Liver has the largest number of residential macrophages, named Kupffer cells, accounting for approximately 20% of the non-parenchymal cells in the liver. In general conditions, Kupffer cells are located in the hepatic sinusoid in order to fully interact with various molecules and cells in the blood. Their main functions are phagocytosing, secreting cytokines [such as IL-1, IL-6, IL-12, IL-18, TNF-α, and IFN-γ (31)], and processing and presenting antigens (32, 33).

Under a resting circumstance, compared with DCs, Kupffer cells express significantly lower levels of MHC-II and costimulatory molecules (such as B7-1, B7-2, CD40). Therefore, as intrahepatic APCs, their functions are significantly weaker than DCs. Moreover, You et al. (34) have found that steady-state Kupffer cells are able to secrete prostaglandin E2 (PGE2) and 15-deoxy-delta 12,14-PGJ_2_ (15d-PGJ_2_), which can directly inhibit antigen-specific CD4^+^ T cells activation (34, 35). In addition, through animal models, Sass et al. (36) confirmed that high doses of TNF can induce hepatocytes apoptosis, whereas low doses of TNF (pretreatment with 10 μ/kg) have the exact opposite role. After the invasion of endotoxin, Kupffer cells secrete anti-inflammatory cytokines such as IL-10 (37) and TGF-β (38) in response to continuous stimulation with low levels of lipopolysaccharide (LPS). This phenomenon is called LPS tolerance. In addition, increased TLR4-mediated expression of adhesion molecules in LSECs and KCs following continuous LPS exposure promoted trapping of T cells within the liver, resulting in lower numbers of circulating primed CD8^+^ T cells and weak immune responses (39).

Apart from secreting cytokines, Kupffer cells can directly interact with Tregs to stimulate their proliferation and induce them to secrete IL-10, which can inhibit the immune effect of cytotoxic T lymphocyte (CTL) on hepatocyte-expressed antigens (40, 41). Kuniyasu et al. (42) have undertaken the researches into the outcomes of activated CD8^+^ T cells in the liver and found that some of them can be retained in the liver. In the meantime, Kupffer cells can encourage the proliferation of these activated CD8^+^ T cells in the early period while they promote apoptosis in the late period. Ultimately, the CTL-mediated cellular immunity can be inhibited by Kupffer cells (42). Through animal experiments, Chen et al. (43) have found that Kupffer cells can promote T cell apoptosis through the Fas/Fas-L pathway. This effect can be inhibited by the Fas-L antibody (43). Similarly, there were researches that showed that Kupffer cells highly expressing PD-L1 can also inhibit the proliferation and functions of T cells by directly contacting them (44). Apart from this, human Kupffer cells activated through TLR2/4 pathways can synthesize IL-10, thus, suppressing IL-18-dependent NK cell activation (45).

## Liver sinusoidal endothelial cells (LSECs) in tolerance

Liver sinusoidal endothelial cells account for about 50% of the non-parenchymal cells in the liver. The structure of LSEC is just like a perforated screen plate. They constitute the wall of the hepatic sinusoid. There is no basement-membrane at the inside/outside of LSECs, with only a few reticular fibers attached on them. In the liver, it has been reported that LSECs can be a unique population of organ-resident APCs; they display scavenger activity with the capacity to (cross) present exogenous antigens on both MHC II and MHC I molecules to CD4 or CD8 T cells, respectively (46, 47). Some animal experiments showed that, in normal conditions, although LSECs express only a few of the MHC class II molecules, they can induce the activation of naive CD4+ T cells and promote their secretion of interferon-γ IFN-γ, IL-4, and IL-10 (48). However, LSECs are not capable of inducing the differentiation of naïve CD4+ T cells into their effector T helper 2 (Th1) cell subpopulation (47).

As APCs, LESCs have specific immune tolerance-inducing effects on both CD4+ and CD8+ T cells. For CD4+ T cells, animal experiments have proved that after injecting allogeneic cells into the portal venous systems, the LSECs in the recipient mice express significantly increased levels of FasL. By this way, LESCs can induce apoptosis of CD4+ T cells via the Fas-FasL pathway, inhibiting the proliferation of CD4+ T cells and their secretion of IL-2 (49). For CD8+ T cells, there is a process called cross-tolerance. As observed with other APCs, LSECs are able to cross-present exogenous antigens to CD8+ T cells via MHC-I molecules. Once LSECs contacted CD8+ T cells, in the early period, CD8+ T cells showed a prominent proliferation effect, and the expression of CD69, CD25, CD44, and PD-1 on the surface of CD8+ T cells were unregulated. But soon afterwards, the interaction between LSECs and CD8+ T cells results in an upregulation of the inhibitory molecules (such as B7-H1) on LSECs, rather than the costimulatory molecules (such as CD80/86). It has been indicated that Bim, a pro-apoptotic Bcl-2 family member, is a pro-apoptotic mediator of this cross-tolerance process (50). Finally, we can obtain a specific subpopulation of LSECs (B7-H1^high^CD80/86^low^LSECs), which can inhibit the cytotoxic effect of CD8+ T cell but fail to induce the clonal deletion of this subpopulation (51).

Besides the mechanism mentioned above, LESCs also have immune tolerance effects that are independent of their antigen presenting function. Through animal experiments, Kruse et al. (52) found that, under the influence of LSECs, CD4+ T cells can differentiate into CD25^low^FoxP3^−^Tregs, which can inhibit the proliferation of naïve CD4+ T cells. The animal model of T cell-mediated autoimmune hepatitis has shown that CD25^low^FoxP3^−^ Tregs can downregulate the alanine aminotransferase (ALT; glutamic-pyruvic transaminase) level in peripheral blood and inhibit the infiltration of inflammatory cells in the liver tissue (52). In terms of immune regulation, LESCs can secrete liver and lymph node sinusoidal endothelial cell C-type lectin (LSECtin). The LSECtin can interact with CD44 on the surface of activated T cells, inhibits the secretion of effector cytokines by them (such as IL-2, IFN-γ and so on), and even induces T cell apoptosis (53). After repetitive invasions of LPS, LSECs reduce NK-κB activation and mediate liver tolerance to maintain the homeostasis in the liver (39). In addition, LESCs can interfere with the activation of CD8+ T cells by DCs through direct physical contact with DCs (54).

## Natural killer cells (NK cells) in tolerance

Natural killer cells account for the largest proportion (about 30–50%) of all lymphocytes (which consist of NK, NKT, γδT, αβT, and B cells) in a normal adult liver (55, 56). They are important mediators of liver damage in viral and inflammatory liver disease (57–59). Their functions are controlled by balance of activatory and inhibitory signals. In general, NK cells can be divided in two groups based on the molecular markers on their cell surface, CD3^−^CD56^dim^CD16^+^CD27^−^ and CD3^−^CD56^bright^CD16^−^CD27^+^ NK cells. Among them, the former performs as professional cytotoxic cell, while the latter has a strong ability of cytokine secretion (mainly IFN-γ and TNF-α) (55, 60). Liver-derived NK cells are different from those in the peripheral blood, with higher expression of activation receptors (such as CD69, NKp44, NKp46, NKG2D), inhibition receptors (such as NKG2A+), and TNF related apoptosis inducing ligand (TRAIL), higher secretion of perforin and granzyme, and stronger cytotoxicity (56, 61, 62).

It is generally believed that, in liver transplantation, NK cells play roles in both rejection and tolerance. According to their origins, liver-derived NK cells can be divided in two groups, donor-derived NK cells and recipient-derived NK cells. It has been found that the recipient-derived NK cells play a major role in immune rejection, while the donor-derived NK cells mainly promote immune tolerance (61). Jamil et al. (63) have found that after liver transplantation, recipient NK cells exhibit tolerant phenotypes, with the downregulation of activating receptors and reduced cytotoxicity and cytokine production. Their research also revealed that NK cell tolerance is associated with perturbation of the IL-12/STAT4 signaling pathway, which might be a therapeutic target for clinical practice (63). There are clinical studies that showed that compared with the recipients who developed post-operation immune rejection, those with post-operation immune tolerance had significantly higher percentages and absolute counts of NK cells in their peripheral blood (64). Li et al. (65) have found that there are 13 genes that are significantly overexpressed in the NK cells of immune tolerant recipients after liver transplantation. This phenomenon suggested that NK cells may be involved in the induction of immune tolerance (65).

## Natural killer T (NKt) cells in tolerance

Natural killer T cells mostly exist in the liver, spleen, and bone marrow. They can express the molecular markers of NK cells. Beyond this, there are specific T cell receptor (TCR) Vα chains on their cell surface, which can recognize the glycolipid antigens presented by CD1d. Through the mechanism mentioned above, NKT cells can rapidly produce a large amount of cytokine (Th1 or Th2 type) and mediate immune response through direct or indirect ways. It is currently believed that Valpha14 NKT cells play a role in graft immune tolerance. Ikehara et al. (66) used animal experiments to show that mice deficient in Valpha14 NKT cells were not able to induce immune tolerance after pancreatic islets transplantation until the exogenous Valpha14 NKT cells were injected into the mice (66).

## Clinical relevance of tolerance in liver transplantation

As compared with other solid organs, it is clear that human liver allografts show unique immunological features. It can be performed across positive cross matches, with small doses of immunosuppressive regimens and less frequent rejection events. For liver transplantation, some patients can eventually totally withdraw their immunosuppression therapy without undergoing rejection. This is a phenomenon called spontaneous operational tolerance, firstly reported in the early 1990s in Pittsburgh (67–69). Following this, several single centers described their experiences with the discontinuation of immunosuppression because of patient non-compliance (70–75). A US multicenter pediatric trial enrolled 20 recipients of parental living donor liver grafts at least 4 years after transplantation (76). Drug withdrawal was successfully accomplished in 12 patients for more than 6 years. Successful drug withdrawal was defined as 1 year off immunosuppression with normal liver function tests, for example liver biopsies performed at enrollment and 2 and 4 years after complete drug discontinuation that failed to show clinically significant histological changes. From these researches, we can see that the time after transplantation is a key factor associated with spontaneous operational tolerance. At present, a number of clinical trials are put into practice to confirm the high prevalence of tolerance among long-term surviving recipients, to help clarify the mechanisms behind human liver allograft tolerance and to determine whether it is possible to prospectively identify tolerant recipients on immunosuppression by employing diagnostic biomarkers.

## Conclusions and future challenges

**Figure 1:**
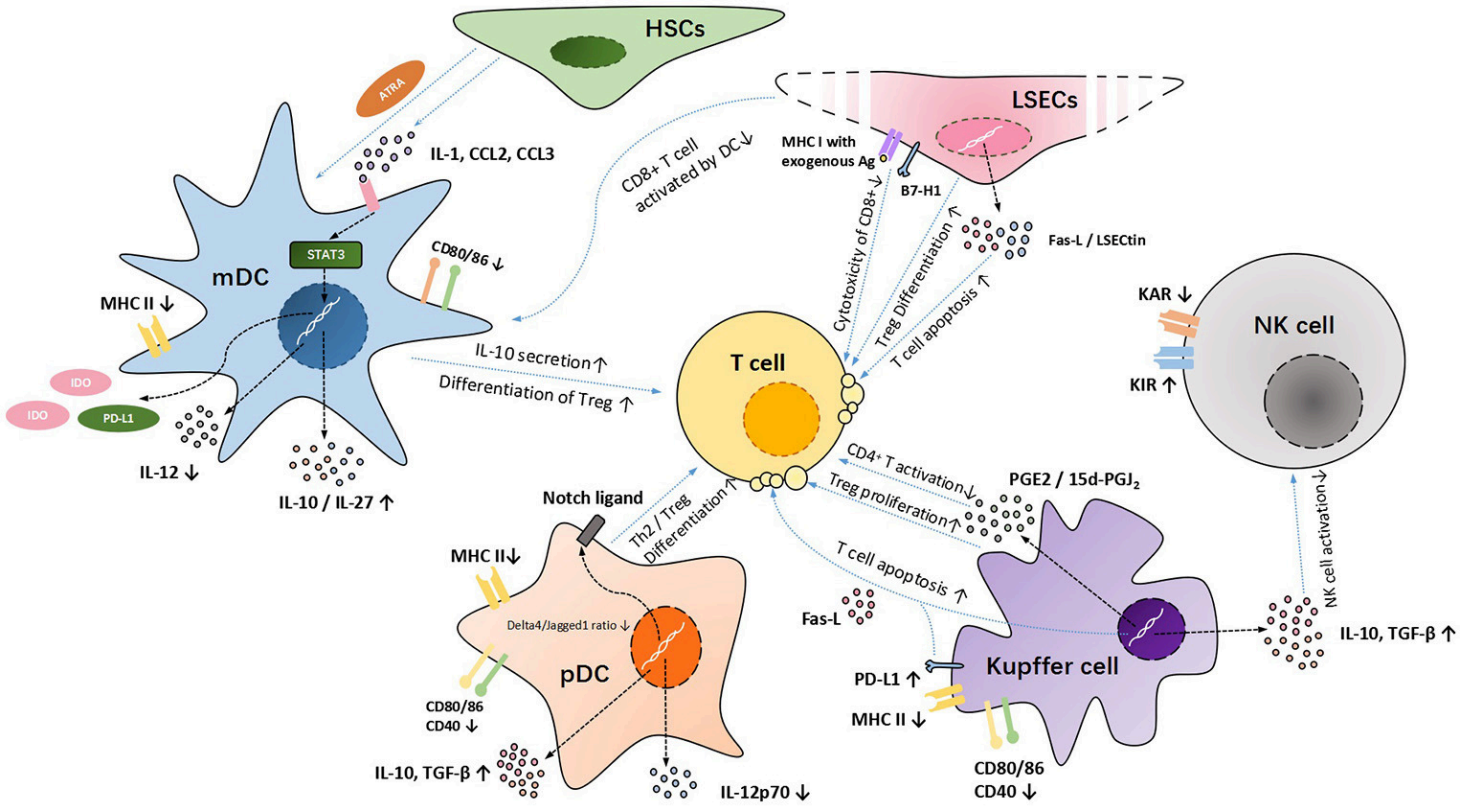
The mechanisms of immune cell tolerance after LTs.

Up to now, it has been more than 40 years since the first time people regarded the liver as an immune-tolerant organ (77). During this period, people researched for the molecular mechanisms inducing intrahepatic immune tolerance and accumulated substantial evidence through animal experiments. However, currently, the relative lack of human studies in this field is one of the major problems that need to be overcome in the future. Although intrahepatic innate immune cells play important roles in inducing immune tolerance after liver transplantation (Figure 1), we seldom hear about clinical cases using their functions to reduce the doses of immunosuppressive agents. In addition, some animal experiments and clinical studies have shown that after the invasion of external pathogens (bacteria or viruses), some receptors (especially TLRs) on the surface of innate immune cells are activated, inducing the release of inflammatory cytokines (such as IL-6, TNF-α, IFN-I, and so on), further interrupting the development of intrahepatic immune tolerance (78). Interestingly, in 2014, the study performed by Bohne et al. (79) showed that for hepatitis C virus (HCV)-infected recipients, tolerance was associated with intrahepatic overexpression of type I IFN and an expansion of exhausted HCV-specific circulating CD8^+^ T cells. This is in contrast to what we had previously reported, that is, IFN-I can activate STAT4 and contribute to Th 1 response, which play a central role in allograft rejection (80). For this special circumstance of HCV infection, the author hypothesized that high intrahepatic IFN-I signaling induced by HCV infection caused T cell exhaustion, inactivated allo-specific T cell clones, and promoted a tolerogenic liver microenvironment that facilitates operational tolerance. On the basis of these results, we conclude that usually intrahepatic innate immune cells are in balanced states, between pro- and anti-inflammation. Under normal resting conditions, intrahepatic innate immune cells mainly induce intrahepatic immune tolerance, whereas under inflammatory conditions, they promote immune response and release inflammatory cytokines. Further exploration of the essential factors in the transformation of intrahepatic innate immune cells between these two states is of great importance for finding out the mechanisms of intrahepatic immune tolerance.

## Author contributions

All authors listed have made a substantial, direct and intellectual contribution to the work, and approved it for publication.

### Conflict of interest statement

The authors declare that the research was conducted in the absence of any commercial or financial relationships that could be construed as a potential conflict of interest.
